# Pharmacokinetic/pharmacodynamic model-based optimization of temocillin dosing strategies for the treatment of systemic infections

**DOI:** 10.1093/jac/dkae243

**Published:** 2024-07-20

**Authors:** Wisse van Os, Alina Nussbaumer-Pröll, Anh Duc Pham, Gert-Jan Wijnant, Perrin Ngougni Pokem, Françoise Van Bambeke, J G Coen van Hasselt, Markus Zeitlinger

**Affiliations:** Department of Clinical Pharmacology, Medical University of Vienna, Waehringer Guertel 18-20, 1090 Vienna, Austria; Department of Clinical Pharmacology, Medical University of Vienna, Waehringer Guertel 18-20, 1090 Vienna, Austria; Division of Systems Pharmacology & Pharmacy, Leiden Academic Centre for Drug Research, Leiden University, Einsteinweg 55, 2333 CC Leiden, The Netherlands; Pharmacologie cellulaire et moléculaire, Louvain Drug Research Institute, Université catholique de Louvain, Avenue E. Mounier 73/B1.73.05, 1200 Brussels, Belgium; Pharmacologie cellulaire et moléculaire, Louvain Drug Research Institute, Université catholique de Louvain, Avenue E. Mounier 73/B1.73.05, 1200 Brussels, Belgium; Pharmacologie cellulaire et moléculaire, Louvain Drug Research Institute, Université catholique de Louvain, Avenue E. Mounier 73/B1.73.05, 1200 Brussels, Belgium; Division of Systems Pharmacology & Pharmacy, Leiden Academic Centre for Drug Research, Leiden University, Einsteinweg 55, 2333 CC Leiden, The Netherlands; Department of Clinical Pharmacology, Medical University of Vienna, Waehringer Guertel 18-20, 1090 Vienna, Austria

## Abstract

**Background:**

Temocillin is increasingly considered as an alternative to carbapenems. However, there is no consensus on optimal dosing strategies and limited data on temocillin efficacy in systemic infections.

**Objectives:**

We compared temocillin dosing strategies using pharmacokinetic/pharmacodynamic (PK/PD) modelling and simulation based on plasma exposure and *in vitro* time–kill data.

**Methods:**

Temocillin effects on four *Escherichia coli* strains were evaluated using static time–kill experiments and the hollow-fibre infection model, in which unbound plasma concentrations following intermittent and continuous infusion regimens of 4 and 6 g daily were replicated over 72 h. A PK/PD model was developed to describe the time–kill data. The PK/PD model was coupled to a population PK model of temocillin in critically ill patients to predict bacterial killing and resistance development following various dosing regimens.

**Results:**

Amplification of resistant subpopulations was observed within 24 h for all strains. The PK/PD model described the observed bacterial kill kinetics and resistance development from both experimental systems well. Simulations indicated dose-dependent bacterial killing within and beyond the currently used daily dose range, and a superiority of continuous compared with intermittent infusions. However, regrowth of resistant subpopulations was frequently observed. For two strains, bacteriostasis over 72 h was predicted only with doses that are higher than those currently licensed.

**Conclusions:**

Continuous infusions and 6 g daily doses of temocillin kill *E. coli* more effectively than 4 g daily doses and intermittent infusions, and may increase efficacy in the treatment of systemic infections. However, higher daily doses may be required to suppress resistance development.

## Introduction

The spread of ESBL-producing Enterobacteriaceae has led to increased carbapenem usage and a subsequent rise in carbapenem-resistant Gram-negative pathogens.^1,2^ Temocillin is a penicillin antibiotic with stability against most β-lactamases, including most ESBL types and AmpC,^3–5^ and thus has carbapenem-sparing potential. Temocillin has a narrow spectrum of activity, which is almost exclusively limited to Enterobacteriaceae and does not include Gram-positive bacteria, anaerobes or *Pseudomonas aeruginosa*.^5^ It is approved in individual, mainly European countries for the treatment of septicaemia, urinary tract infections (UTIs) and lower respiratory tract infections where susceptible Gram-negative pathogens are suspected or confirmed.^6^

Data on the pharmacokinetics (PK) and pharmacodynamics (PD) of temocillin, as well as high-quality clinical efficacy data, are scarce and consensus on optimal dosing regimens is lacking. In 2019, EUCAST published temocillin breakpoints, categorizing all isolates with MICs up to 16 mg/L as ‘susceptible, increased exposure’. EUCAST recommends that only a 2 g q8h regimen should be used, instead of the standard 2 g q12h regimen, to cover the entire WT distribution of relevant pathogens.^7^ EUCAST additionally noted these recommendations apply to complicated UTIs and urosepsis only, as there are insufficient data to recommend breakpoints and dosing regimens for other infection types.^7^ Since then, various groups have reported retrospective data indicating good clinical efficacy (>85%) of temocillin for the treatment of UTIs with daily doses of 4 g,^8–12^ as previously recommended by national guidelines.^13^ These high efficacy rates may be explained by the accumulation of temocillin in urine,^14^ resulting in high exposure at the infection site. For other infection types, however, cure rates were generally lower than those for UTIs in the same cohort.^8–10,15^ One retrospective trial with 54% non-UTI cases found that a 1 g q12h regimen resulted in significantly worse outcomes compared with a 2 g q12h regimen,^16^ but it is unclear whether this apparent dose-dependent efficacy extends to higher doses. In addition to increasing the dose, administering temocillin as a continuous infusion (CI) may be advantageous since β-lactams exert time-dependent antibiotic activity.^17,18^

In this study, we applied PK/PD modelling and simulation to optimize temocillin dosing strategies for the treatment of systemic infections. To this end, we integrated *in vitro* data obtained in static time–kill (STK) experiments and the hollow-fibre infection model (HFIM), and the population PK of temocillin in critically ill patients.

## Materials and methods

### Bacterial strains

Experiments were performed with four *Escherichia coli* strains: ATCC 25922 and three strains isolated at the Vienna General Hospital, two of which were ESBL producers (Table 1). The strains were selected based on their temocillin MIC values, which covered the less susceptible end of the WT distribution for *E. coli* (4–16 mg/L).^19^ MIC values were determined in triplicate by broth microdilution in CAMHB (Sigma–Aldrich, Austria) following CLSI guidelines.^20^

**Table 1.** Characteristics of the *E. coli* strains used in the study

Strain	Source	ESBL genes	Temocillin MIC (mg/L)
ATCC 25922	Reference strain		16
ISOL_MIC16_	Catheter urine	*bla*_CTX-M-15_, *bla*_OXA-1_	16
ISOL_MIC8_	Skin swab		8
ISOL_MIC4_	Rectal swab	*bla*_TEM-1_, *bla*_CTX-M-1_	4

### STK experiments

The effects of temocillin (Negaban^®^, Eumedica S.A., Belgium, purchased from the Vienna General Hospital pharmacy) were first evaluated in STK experiments. Tubes containing 5 mL of pre-warmed (37°C) CAMHB were inoculated at a target bacterial population of 1.5 × 10^6^ cfu/mL. Inocula were prepared using the 0.5 McFarland standard from a liquid culture that had been incubated for 1 h to ensure the population was in log-phase growth. The STK experiments were performed in triplicate with temocillin concentrations ranging from 0.125 to 8× the MIC of the respective strain, in 2-fold steps, plus a growth control. The tubes were incubated at 37°C in a shaking water bath. Over a period of 24 h, samples were taken, serially diluted in 0.9% saline and plated in 20 μL drops on Columbia agar plates with 5% sheep blood (bioMérieux, France). At selected timepoints, samples were also plated on cation-adjusted Mueller–Hinton agar (Sigma–Aldrich, Austria) containing 32 mg/L temocillin to quantify resistant subpopulations. Colonies were counted after incubation at 37°C in ambient air (24 h for antibiotic-free plates, up to 72 h for temocillin-containing plates). The theoretical limit of detection (LOD) was 50 cfu/mL.

### HFIM

The HFIM was used to evaluate bacterial response to clinically relevant PK profiles. In the HFIM experiments, a dialysis cartridge (FX paed, Fresenius Medical Care, Germany) with semi-permeable Helixone^®^ polysulfone fibres and an extracapillary space volume of 50 mL was connected to a flask (the central compartment) via silicone tubing (Cole-Parmer, USA). The contents of the central compartment were continuously mixed and kept at 37°C using a magnetic stirrer with a thermometer-regulated hot plate. A peristaltic pump (Masterflex^®^ L/S^®^, Cole-Parmer, USA) was used to rapidly (50 mL/min) circulate the contents of the central compartment through the fibres, allowing equilibration with the extracapillary space of the cartridge, where the bacteria were located. To ensure mixing, the contents of the extracapillary space were circulated in the opposite direction using a tubing circuit and a peristaltic pump.^21^ Another peristaltic pump was used to supply fresh CAMHB to the central compartment and pump out its contents into a waste flask at the same rate, thus mimicking drug clearance. Temocillin doses were administered to the central compartment using a syringe pump (SP101IZ, World Precision Instruments, USA). The first drug administration was started immediately after inoculation. The HFIM setup is schematically depicted in Figure S1 (available as Supplementary data at *JAC* Online).

Four IV dosing regimens were replicated over 72 h in the HFIM: (i) 2 g q12h intermittent infusion (II); (ii) 2 g q8h II; (iii) 4 g/day CI with a 2 g loading dose (LD); and (iv) 6 g/day CI with a 2 g LD. Doses were infused over 30 min. To obtain the unbound plasma PK profiles to replicate in the HFIM, deterministic simulations of a temocillin population PK model in critically ill patients were performed and concentrations were multiplied by 0.41 based on the mean protein binding of 59% observed in this population.^22^ Pump rates and other experimental parameters were selected to mimic the simulated PK in the HFIM (Table S1). Two hours after each drug infusion, the rate of the pump governing drug clearance was decreased to mimic the biphasic elimination of temocillin from plasma. For the CI regimens, temocillin was added directly to the media and every 12 h the media inflow bottle was replaced with a freshly prepared one. Inoculum preparation and bacterial count quantification were performed as described for the STK experiments. A sample from the central compartment was plated daily to check for contamination.

### PK assay

To validate the experimental temocillin concentrations and account for potential deviations from the targeted concentrations during PK/PD model development, samples were taken from the central compartment at regular intervals and stored at −80°C. Preliminary experiments showed good agreement between temocillin concentrations in the central compartment and the extracapillary space of the cartridge (Pearson correlation coefficient 0.95; r^2 ^= 0.90; *n* = 16 samples). In addition, samples containing temocillin in CAMHB at target concentrations of 4, 20 and 40 mg/L (*n* = 12 per concentration), prepared from the stock solution used for the STK experiments, were assayed. Total temocillin concentrations were measured using a previously described HPLC-MS/MS method,^23^ which was validated for use with CAMHB as matrix following the relevant FDA guidelines (Supplementary data).^24^

### PK/PD model

**Figure 1.**
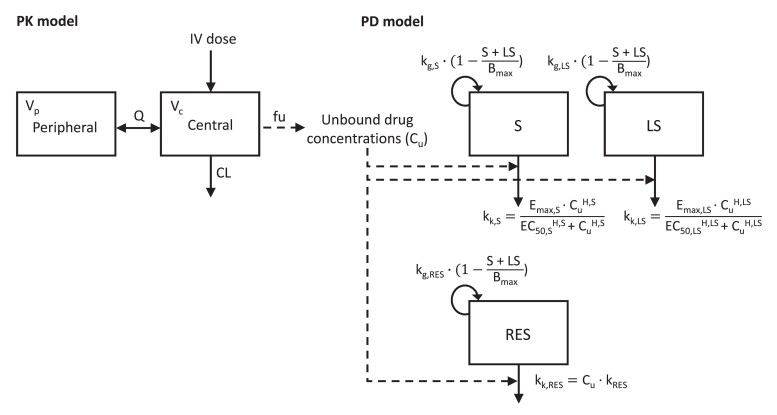
Structure of the temocillin PK/PD model used for the simulations. Solid lines indicate mass transfers; dashed lines indicate relationships between model components. S, susceptible subpopulation; LS, less susceptible subpopulation; RES, resistant subpopulation. The total bacterial population observed on drug-free agar plates is given by the sum of S and LS, and the subpopulation growing on agar containing 32 mg/L temocillin is given by RES. Explanations of other abbreviations are provided in Tables 2 and 3.

A PK/PD model was developed based on the combined STK and HFIM data for each strain. The modelling process consisted of three distinct steps. First, only the total bacterial counts obtained in the STK experiments were modelled in order to obtain preliminary estimates on bacterial growth and concentration–effect relationships informed by rich data across a wide range of drug concentrations. Population growth was described with a first-order rate constant and was limited by the estimated maximum bacterial concentration within the experimental system. Linear, power and (sigmoid) *E*_max_ models were evaluated to describe temocillin concentration–effect relationships. Since heteroresistance was observed in the growth control experiments, observed regrowth or reductions in antimicrobial effects were modelled by dividing the bacterial population into a pre-existing susceptible and less susceptible subpopulation (Figure 1). The M3 method was used to handle observations below the LOD.^25^ Residual unexplained variability (RUV) was described with an additive error model on the log_10_ scale. In the second step, the HFIM time–kill data were added and the model was refined using the data from both setups simultaneously, with the structure and parameter estimates of the models based on the STK data as a starting point. Parameters were re-estimated based on the data from both setups, and we investigated whether the model could be simplified or whether alternative parameterizations improved model fit. In the third step, the observed bacterial counts on agar containing 32 mg/L temocillin were modelled by including another subpopulation. Since this subpopulation also appears on drug-free agar plates, it did not contribute to total bacterial count in the model.

To accurately describe concentration–effect relationships, the PK part of the models was based on the measured temocillin concentrations in the time–kill experiments, rather than the targeted concentrations. To achieve this for the HFIM experiments, a PK model was fitted to the observed concentrations in each experiment. For the II regimens, the volume of distribution was estimated, as well as two clearance values, since two pump rates were used throughout these experiments. For the CI regimens, in addition to the distribution volume and one clearance value to describe the loading dose PK, a steady-state concentration was estimated, corresponding to a baseline concentration since temocillin was added directly to the media.

Model evaluation and selection was based on the objective function value [a decrease of >3.84 points was used as cut-off for statistical significance (α = 0.05) for nested models with one additional degree of freedom], precision and plausibility of parameter estimates, and visual predictive checks (*n* = 1000).

### PK/PD simulations

Bacterial response to various dosing regimens was simulated by replacing the PK part of the developed PK/PD model for each strain with a population PK model of temocillin in critically ill patients.^22^ The parameter estimates of this model are provided in Table 2. Simulated temocillin concentrations were converted to free concentrations using the mean unbound fraction (fu) of 0.41 observed in the PK study. The PK/PD simulations were also performed with fu values of 0.25 and 0.57, corresponding to the mean fu ± one standard deviation.^22^ The Monte Carlo simulations (*n* = 1000 patients per regimen) included the inter-individual variability (IIV) in temocillin PK and not the RUV in the PK or PK/PD models. The initial size of the total bacterial population was set to 10^6^ cfu/mL. The initial size of each subpopulation was scaled accordingly, based on the PK/PD model estimates.

**Table 2.** Pharmacokinetic parameters used for simulations, from Laterre *et al.*^22^

Parameter	Value	IIV (CV, %)^a^
CL (L/h)	3.69	36
*V*_c_ (L)	14.0	58
Q (L/h)	8.45	
*V*_p_ (L)	21.7	
fu	0.25^b^, 0.41, 0.57^b^	

*V*_c/p_, distribution volume of the central/peripheral compartment; Q, intercompartmental CL.

^a^Converted to variances (*ω*^2^) using %CV =  $\sqrt{e^{\omega^{2}}-1}\times 100$.

^b^Results shown in the Supplementary data.

### Software

Modelling and simulation were performed with NONMEM 7.4 (ICON plc, USA) using Laplacian estimation, in combination with PsN (v5.3.0; Uppsala University, Sweden)^26^ and Piraña (v21.11.1; Certara, USA).^27^ R (v4.2.2) was used for dataset preparation, processing model output and visualizations.^28^

## Results

### Time–kill experiments

**Figure 2.**
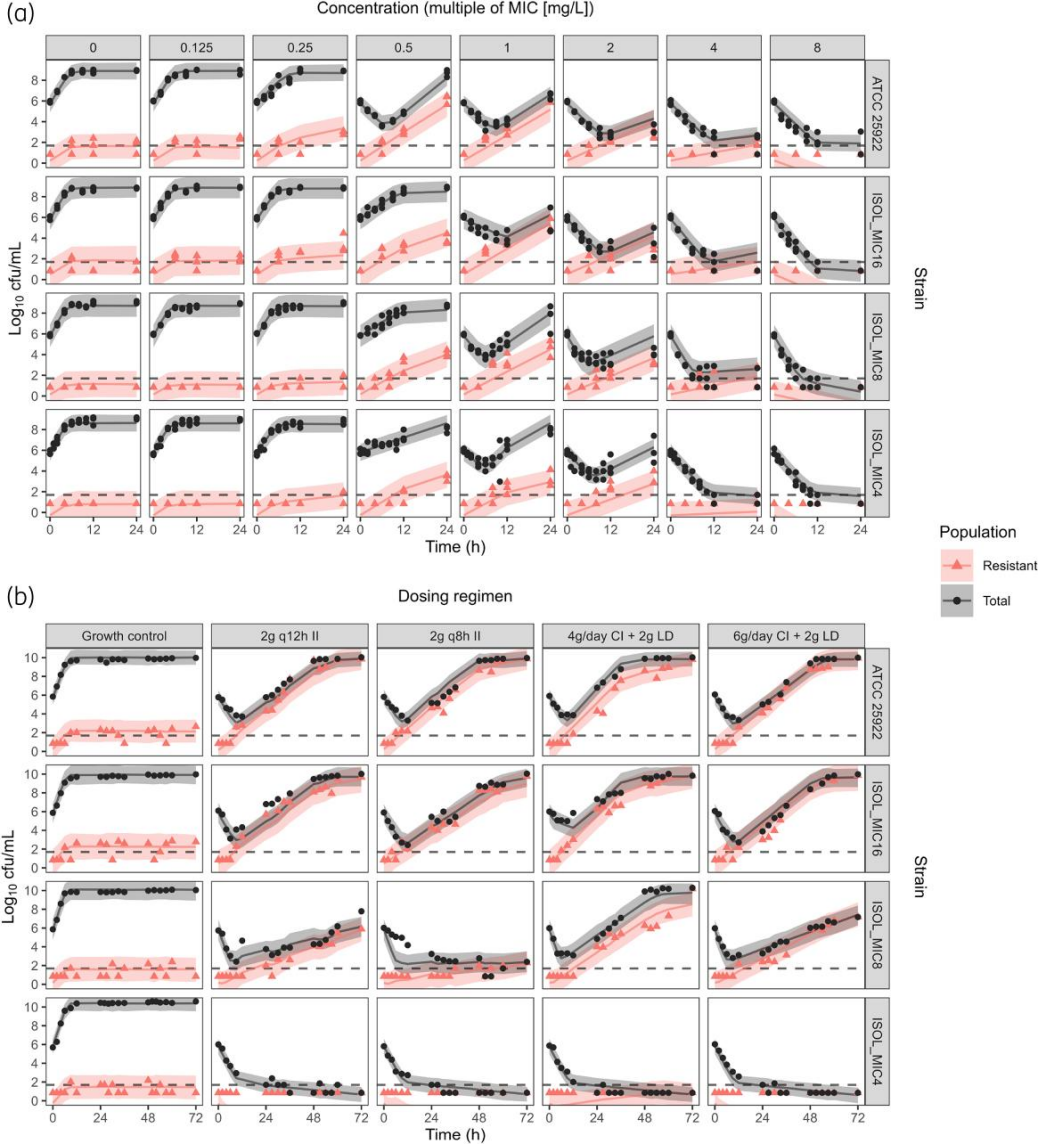
Visual predictive check to evaluate model fit to the observations in the STK (a) and HFIM (b) experiments. Symbols represent observations for the total bacterial population (black dots) and the subpopulation growing on agar containing 32 mg/L temocillin (red triangles). Solid lines represent the median values of model simulations and the shaded areas the 95% prediction intervals. The dashed line indicates the LOD (50 cfu/mL). Observations below the LOD are plotted at (log_10_ LOD)/2. This figure appears in colour in the online version of *JAC* and in black and white in the print version of *JAC*.

In the STK experiments, little temocillin effect was observed at concentrations up to 0.25× MIC (Figure 2). Amplification of resistant subpopulations able to grow on agar containing 32 mg/L temocillin was observed already at subMIC concentrations, but was suppressed at 4–8× MIC.

Regrowth following multiple log-reductions in bacterial count was observed in all HFIM experiments with ATCC 25922 and ISOL_MIC16_ and in all but one with ISOL_MIC8_. Sustained reductions in bacterial count were only achieved for ISOL_MIC4_. As in the STK experiments, regrowth in the HFIM was associated with the amplification of resistant subpopulations.

### PK validation

The samples spiked with temocillin concentrations of 4, 20 and 40 mg/L contained on average 78.1% [coefficient of variation (CV) 21.2%] of the targeted concentrations. Thus, to accurately inform concentration–effect relationships during PK/PD model development, the target concentrations in the STK experiments were multiplied by 0.781.

In HFIM experiments in which II regimens were replicated, measured temocillin concentrations were generally higher than the targeted concentrations, particularly around the *C*_max_ (Figure S2). Conversely, when CI regimens were replicated, measured concentrations were consistently below the target concentration. The PK models that were developed for each HFIM experiment to account for these deviations described the observed temocillin concentrations well (Figure S2). Temocillin degradation was not observed in the CI experiments, in line with reports on the stability of temocillin at 37°C.^17,29,30^

### PK/PD model

For all strains, temocillin effects on the total bacterial population were best described using (sigmoidal) *E*_max_ models. Separate growth rates and drug-effect parameters for the susceptible and less susceptible subpopulations were identifiable (Equation 1, Equation 2). The models developed on STK data alone were able to predict the first 12–24 h of HFIM observations, but did generally not accurately predict observations at later timepoints (Figure S3). Parameter re-estimation and minor model modifications, i.e. estimating the Hill (H) coefficient in the *E*_max_ function describing temocillin effect against the less susceptible subpopulation for ISOL_MIC4_ and ISOL_MIC8_ and fixing it to 1 for ISOL_MIC16_, resulted in models that fitted the data from both experimental setups well (Figure 2). If the estimate of the Hill coefficient was ≥10, it was fixed to 10, as higher values had little impact on model fit due to the steepness of the concentration–effect relationship at this value and were associated with poor parameter precision. The parameter representing the maximum bacterial population was estimated separately for the STK and HFIM data, since it was observed to be approximately 10-fold higher in the HFIM cartridge than in the tubes used for STK experiments. Temocillin effect on the resistant subpopulation growing on agar with 32 mg/L temocillin was described with a linear function for all strains (Equation 3); more complex effect models were not supported by the data. Parameter estimates and definitions are listed in Table 3. The NONMEM code is provided in the Supplementary data.


(1) $$\frac{dS}{dt}=k_{g,S\;}\cdot \left(1-\frac{S+LS}{B_{max}}\right)\cdot S-\frac{E_{max,S\;}\cdot \;C^{H,S}}{E{C_{50,S}}^{H,S}+C^{H,S}}\cdot S$$

(2) $$\frac{dLS}{dt}=k_{g,LS\;}\cdot \left(1-\frac{S+LS}{B_{max}}\right)\cdot LS-\frac{E_{max,LS\;}\cdot \;C^{H,LS}}{E{C_{50,LS}}^{H,LS}+C^{H,LS}}\cdot LS$$

(3) $$\frac{dRES}{dt}=k_{g,RES\;}\cdot \left(1-\frac{S+LS}{B_{max}}\right)\cdot RES-k_{lin,RES}\cdot C\cdot RES$$

**Table 3.** Parameter estimates of the pharmacodynamic model for the four strains

Parameter	Unit	Description	Estimate (%RSE)			
			ATCC 25922	ISOL_MIC16_	ISOL_MIC8_	ISOL_MIC4_
cfu_t0,S_	log_10_ cfu/mL	Initial population size (S)	5.75 (1.1)	5.83 (1.3)	5.79 (1.5)	5.78 (0.9)
k_g,S_	h^−1^	Growth rate constant (S)	1.29 (5.5)	1.39 (7.0)	1.51 (7.9)	1.47 (5.8)
*E*_max,S_	h^−1^	Maximum drug effect rate constant (S)	2.06 (3.3)	2.38 (4.3)	2.86 (4.5)	2.38 (3.5)
EC_50,S_	mg/L	Drug concentration at which effect is half-maximal (S)	3.56 (1.9)	8.31 (4.7)	3.75 (4.9)	1.57 (3.3)
H,_S_	—	Hill coefficient (S)	10 (FIX)	2.45 (9.0)	3.51 (19.0)	2.73 (12.8)
cfu_t0,LS_	log_10_ cfu/mL	Initial population size (LS)	1.30 (12.1)	0.837 (31.2)	2.09 (5.6)	2.08 (5.4)
k_g,LS_	h^−1^	Growth rate constant (LS)	0.797 (7.5)	0.795 (11.3)	0.720 (19.6)	0.796 (3.0)
*E*_max,LS_	h^−1^	Maximum drug effect rate constant (LS)	0.777 (9.2)	1.09 (10.1)	1.01 (22.6)	0.843 (2.5)
EC_50,LS_	mg/L	Drug concentration at which effect is half-maximal (LS)	17.1 (9.1)	37.0 (43.5)	17.1 (10.6)	6.34 (1.1)
H,_LS_	—	Hill coefficient (LS)	1.68 (12.1)	1 (FIX)	1.79 (28.3)	10 (FIX)
cfu_t0,RES_	log_10_ cfu/mL	Initial population size (RES)	0.237 (46.4)	0.528 (21.4)	0.156 (87.8)	−0.271 (47.2)
k_g,RES_	h^−1^	Growth rate constant (RES)	0.583 (3.8)	0.589 (4.2)	0.510 (4.5)	0.558 (3.7)
k_lin,RES_	L/mg·h^−1^	Linear drug effect rate constant (RES)	0.00867 (9.8)	0.00940 (8.8)	0.0137 (5.7)	0.0421 (3.6)
B_max,STK_	log_10_ cfu/mL	Maximum bacterial density in STK experiments	8.89 (0.9)	8.86 (0.9)	8.71 (0.9)	8.63 (0.7)
B_max,HFIM_	log_10_ cfu/mL	Maximum bacterial density in HFIM experiments	10.0 (0.7)	9.94 (1.0)	10.1 (1.3)	10.4 (1.0)
RUV_total_	log_10_ cfu/mL	Additive residual variability (S + LS) (standard deviation)	0.403 (4.6)	0.507 (4.6)	0.555 (4.5)	0.413 (4.4)
RUV_RES_	log_10_ cfu/mL	Additive residual variability (RES) (standard deviation)	0.614 (7.0)	0.711 (6.8)	0.655 (8.6)	0.638 (11.9)

RSE, relative standard error; S, susceptible bacterial subpopulation; LS, less susceptible bacterial subpopulation; RES, resistant bacterial subpopulation growing on agar containing 32 mg/L temocillin.

### Simulations

**Figure 3.**
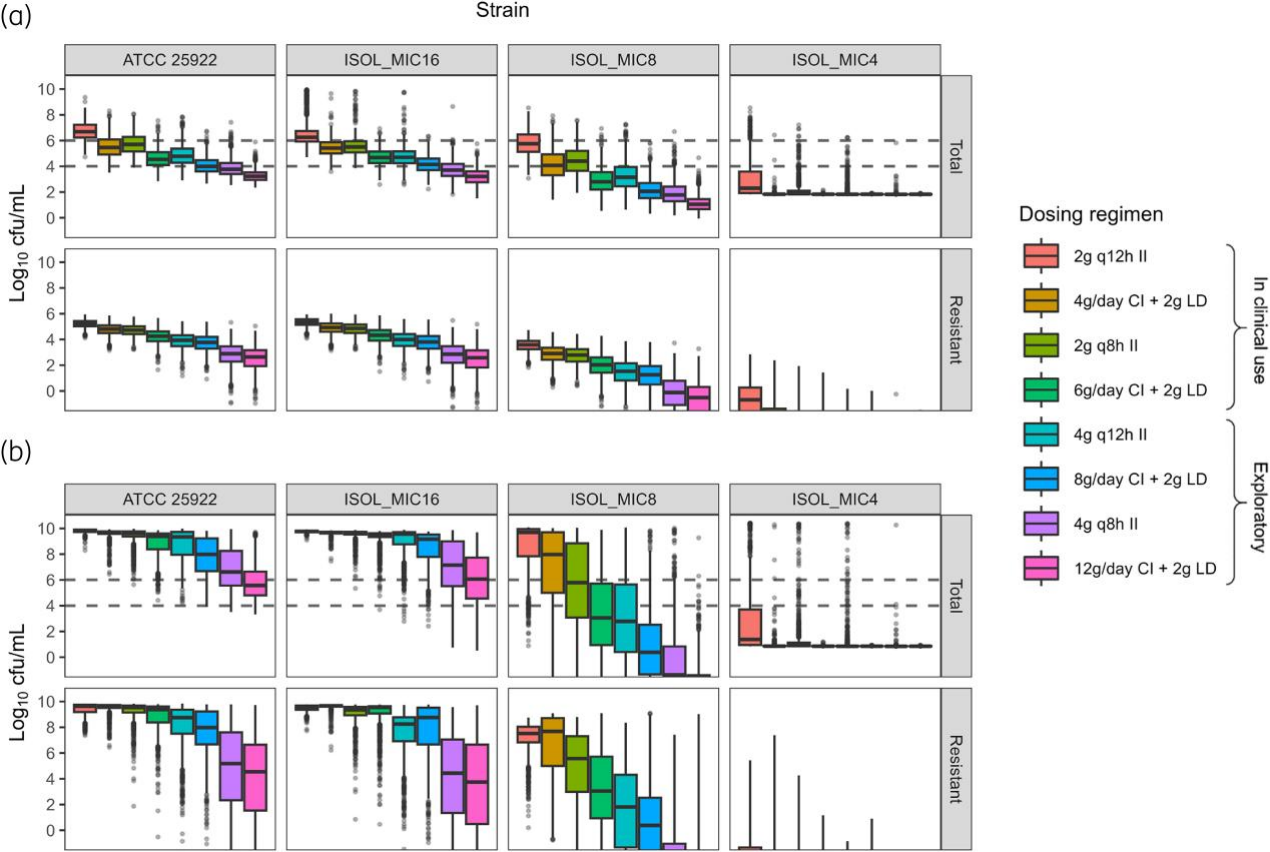
Predicted bacterial counts of the total population (top panels) and the population growing on plates with 32 mg/L temocillin (bottom panels) at 24 h (a) and 72 h (b) following different dosing regimens and unbound fraction of 0.41. In each panel, the four leftmost boxplots represent dosing regimens currently in clinical use, while the four rightmost boxplots represent alternative regimens. The horizontal dashed lines correspond to the initial size of the total bacterial population (10^6^ cfu/mL) and a 2 log_10_ reduction in bacterial count. This figure appears in colour in the online version of *JAC* and in black and white in the print version of *JAC*.

The predicted total and resistant bacterial population sizes at 24 and 72 h for various dosing regimens and fu of 0.41 are depicted in Figure 3. The full PK and PD time courses are shown in Figure S4. For ATCC 25922 and ISOL_MIC16_, both with temocillin MICs of 16 mg/L, the median predicted bacterial count at 24 h was below the stasis level for all regimens apart from the 2 g q12h regimen. However, doses that are currently not licensed^6^ were required to achieve 2 log_10_ reductions in bacterial count over 24 h. Full regrowth of a resistant population was predicted at 72 h in the majority of simulations for all currently used regimens, and the median predicted total bacterial count for these two strains reached the stasis level only with a 12 g daily CI regimen. Median predicted bacterial counts of ISOL_MIC8_ were below the stasis level at 24 h for all simulated regimens. Median predicted stasis at 72 h was achieved for 6 g daily regimens, but not for 4 g daily regimens. A 6 g daily CI regimen or daily dosages ≥8 g were required to achieve 2 log_10_ reductions over 72 h for this strain. All simulated dosing regimens resulted in sustained killing of ISOL_MIC4_. Except for the 2 g q12h regimen, near-maximum cfu reductions given the parameter estimates for this strain were achieved in most simulations, although the variability in response at equivalent daily dosages was larger for II regimens.

The impact of different fu values on the simulation results is depicted in Figure S5. As expected, the observed bacterial counts decreased as the fu increased. The general trend, however, was similar for all fu values: continuous infusion regimens resulted in lower bacterial counts than intermittent infusion regimens, and bacterial killing was mostly dose-dependent within the simulated dose range.

## Discussion

Due to the scarcity of data on temocillin PK/PD and clinical efficacy, there is no consensus on optimal dosing strategies.^8–11,31^ This study aimed to compare the effect of various dosing regimens using PK/PD modelling and simulation, combining *in vitro* time–kill data and the plasma PK of temocillin in critically ill patients.

Our results indicate that temocillin effects against *E. coli* are dose-dependent within the currently used daily dose range of 4–6 g, and higher. Several retrospective studies indicated that dosing temocillin at 6 g daily is safe, but reported no statistically significant differences in clinical outcomes between 4 and 6 g daily dosages.^8,10,15^ However, the limitations inherent to retrospective studies, as well as the limited sample sizes with few treatment failures, may have concealed an effect. Moreover, the majority of patients included in these studies were treated for UTIs or bacteraemia of urinary origin. The potential benefit of higher dosages may not apply to these indications since temocillin accumulates in urine^14^ and cure rates are overall high. Our results also suggest that CI regimens of temocillin kill bacteria more effectively than II regimens. The stability of temocillin at temperatures up to 37°C makes it suitable for CI.^17,29,30^ This mode of administration may appeal to clinicians who, in the absence of robust clinical evidence supporting 6 g daily dosing, stick to 4 g daily dosages, e.g. to minimize antibiotic usage or for financial reasons.^9–11^

The results of this study are in accordance with PTA analyses that suggested better coverage of temocillin with 6 g compared with 4 g daily dosages,^17^ and with CI compared with II regimens.^18,32^ PTA analyses commonly use 40%–50% *fT*_>MIC_ as PK/PD target for temocillin, which for other penicillins is associated with bacteriostasis over 24 h in the neutropenic murine thigh infection model.^33^ PTA analyses using this PK/PD target underpinned the EUCAST recommendation to dose temocillin at 2 g q8h instead of 2 g q12h, since it increased the PTA at an MIC of 16 mg/L.^7^ Our PK/PD simulations are broadly in line with these findings, as dosing 2 g q8h compared with q12h increased the probability of bacterial density being below the stasis level at 24 h for the strains with temocillin MICs of 16 mg/L.

However, looking beyond the 24 h timepoint, net increases in bacterial population size were frequently predicted with the currently used regimens. Based on the median values of our simulations, only a 12 g daily CI regimen suppressed regrowth to the bacteriostasis level for all strains over 72 h. This suggests higher doses may be required in some cases, e.g. in immunocompromised patients or when targeting strains with temocillin MIC values close to the resistance breakpoint. Doses above 6 g daily are currently not licensed or recommended.^6^ There are limited data on safety and toxicity of temocillin. Daily doses up to 8 g were safe in healthy volunteers,^6,34^ and in animal studies doses up to 1000 mg/kg were well tolerated.^35^ However, the safety of temocillin at increased doses would have to be closely monitored if used in selected patients. The observed regrowth was accompanied by amplification of subpopulations phenotypically resistant to temocillin. It should be noted that antibiotic resistance may develop more readily *in vitro* than clinically.^36^ Nevertheless, cases of emerging resistance during temocillin treatment, also at 6 g daily, have been reported.^12^ The risk of resistance development during temocillin treatment, particularly in immunocompromised patients, should be evaluated in future studies.

Through pharmacometric modelling, we integrated data from STK and HFIM experiments, thereby leveraging the advantages of both experimental setups. STK experiments were performed in triplicate and using a wide range of temocillin concentrations, thus providing rich data to estimate concentration–effect relationships. The HFIM is resource-intensive, meaning a limited number of experiments can feasibly be performed, but enables the following of bacterial response to dynamic, clinically relevant drug concentrations over longer time periods. The PK/PD model based on the STK data alone was generally able to predict the bacterial response observed in the HFIM during the first 12–24 h, as observed by others.^37^ However, the regrowth was not always predicted accurately, presumably because the parameters describing it are informed by relatively few observations in the STK experiments. Extending the duration of STK experiments or taking additional samples between 12 and 24 h may improve the predictive performance of models based on STK data over longer time periods and reduce the need for time-consuming and resource-intensive HFIM experiments.

A strength of the PK/PD modelling approach used in this study is that it considers the time courses of PK and PD simultaneously with continuous bacterial count data over 72 h as the endpoint, enabling a detailed comparison of dosing strategies. This is in contrast to the PK/PD target attainment approach, in which dynamic PK and PD time courses over 24 h are reduced to a threshold value and information on the rate and extent of antibiotic effects is lost. The differences between the two approaches can be illustrated by comparing our simulations with a PTA analysis by Tsakris *et al.*,^18^ which used the same temocillin PK model for simulations.^22^ The authors reported higher PTA for a 6 g dosage administrated as CI (93%) compared with II (87%) at an MIC value of 16 mg/L. At MIC values below 16 mg/L, however, no difference in PTA was observed since all simulated patients reached the PK/PD target.^18^ In the present study we observed a comparative benefit of CI over II also for strains with MIC values below 16 mg/L.

A limitation of this study is that *in vitro* experiments do not reflect the *in vivo* infection site environment, where bacterial growth may be slower^38^ and a (partly) functioning immune response may suppress regrowth after multiple-log reductions in bacterial concentrations. These factors likely contribute to the discrepancy between the frequent regrowth observed in this study and the low microbiological failure rates following temocillin treatment in systemic infections reported in literature.^10,16^ Additionally, clinical outcomes are influenced by patient characteristics and comorbidities beyond antibiotic-induced bacterial killing. For these reasons, PK/PD simulations based on *in vitro* data should not be directly translated to predict antibiotic effects in patients. They are useful, however, for comparing the PK/PD and relative effects of different dosing strategies, particularly when limited efficacy data are available. Another limitation is that temocillin effects were investigated only against *E. coli*; the results of the current study may not apply to other pathogens. Finally, the population PK model by Laterre *et al.*^22^ was developed using data from a small population (*n* = 11). The true variability in temocillin PK in critically ill patients may thus not be reflected in our simulations. Laterre *et al.* also did not observe the saturable protein binding of temocillin reported by others.^32,39^ It should be noted that plasma protein binding of temocillin is associated with high variability.^22,32,39^ Modifying the fu in our PK/PD simulations influenced the observed bacterial counts but overall did not change the comparative performance of the evaluated dosing regimens.

Ideally, prospective trials comparing temocillin dosing strategies for the treatment of systemic infections would be performed to confirm the results of this study. Such trials may not be feasible, however, given the large number of patients that are likely required to show dose–response relationships. Nonetheless, prospective trials evaluating whether temocillin is a valid alternative to carbapenems for systemic infections are needed. One such trial, comparing temocillin dosed at 2 g q8h versus carbapenems for the treatment of bacteraemia due to third-generation cephalosporin-resistant Enterobacterales is currently underway.^40^ Our results support the selection of 6 g instead of 4 g daily doses in such trials.

In conclusion, 6 g daily doses and continuous infusions of temocillin kill *E. coli* more effectively than 4 g daily doses and intermittent infusions and may increase the efficacy of temocillin for treatment of systemic infections. However, higher daily doses may be required to suppress resistance development.

## Supplementary Material

dkae243_Supplementary_Data

## Supplementary data

Figures S1 to S5 and Table S1 are available as Supplementary data at *JAC* Online.
