# Supplementary material for: Pharmacokinetic/pharmacodynamic model-based optimization of temocillin dosing strategies for the treatment of systemic infections
Source: J Antimicrob Chemother. 2024 Jul 20;79(10):2484–92. doi: 10.1093/jac/dkae243 (PMC11442000; doi:10.1093/jac/dkae243)
Supplement: dkae243_Supplementary_Data [file dkae243_supplementary_data.zip › Supplement_revised.docx]

**Supplementary Material**

**Pharmacokinetic-pharmacodynamic model-based optimisation of temocillin dosing strategies for the treatment of systemic infections**

Wisse van Os^1^, Alina Nussbaumer-Pröll^1^, Anh Duc Pham^2^, Gert-Jan Wijnant^3^, Perrin Ngougni Pokem^3^, Françoise Van Bambeke^3^, J.G. Coen van Hasselt^2^, Markus Zeitlinger^1^*

^1^Department of Clinical Pharmacology, Medical University of Vienna, Waehringer Guertel 18-20,
1090 Vienna, Austria.
^2^Division of Systems Pharmacology & Pharmacy, Leiden Academic Centre for Drug Research, Leiden University, Einsteinweg 55, 2333 CC Leiden, The Netherlands
^3^Pharmacologie cellulaire et moléculaire, Louvain Drug Research Institute, Université catholique de Louvain, Avenue E. Mounier 73/B1.73.05, 1200 Brussels, Belgium

* Corresponding author. Tel: +43-(0)1-40400-29800; Fax: +43-(0)1-40400-29980; E-mail: markus.zeitlinger@meduniwien.ac.at

**Table S1.** Experimental parameters selected to replicate clinical PK profiles in the hollow-fibre infection model.

| **Parameter** | **II regimens** | **CI 4g/day + 2g LD** | **CI 6g/day + 2g LD** | **Growth control** |
| --- | --- | --- | --- | --- |
| Volume of circulating media (mL) | 360 | 360 | 360 | 360 |
| Clearance pump rate (mL/min) | 3.47 (0-2 h after dose); 0.67 (other) | 6.93 (0-2 h); 0.67 (>2 h) | 6.93 (0-2 h); 0.67 (>2 h) | 0.67 |
| Temocillin administered per dose (mg) | 19.88 | 13.96 (only LD) | 9.58 (only LD) | - |
| Drug infusion time (min) | 30 | 30 (only LD) | 30 (only LD) | - |
| Temocillin added directly to media, for CI regimens (mg/L) | - | 18.52 | 27.76 | - |

II: intermittent infusion; CI: continuous infusion; LD: loading dose

**
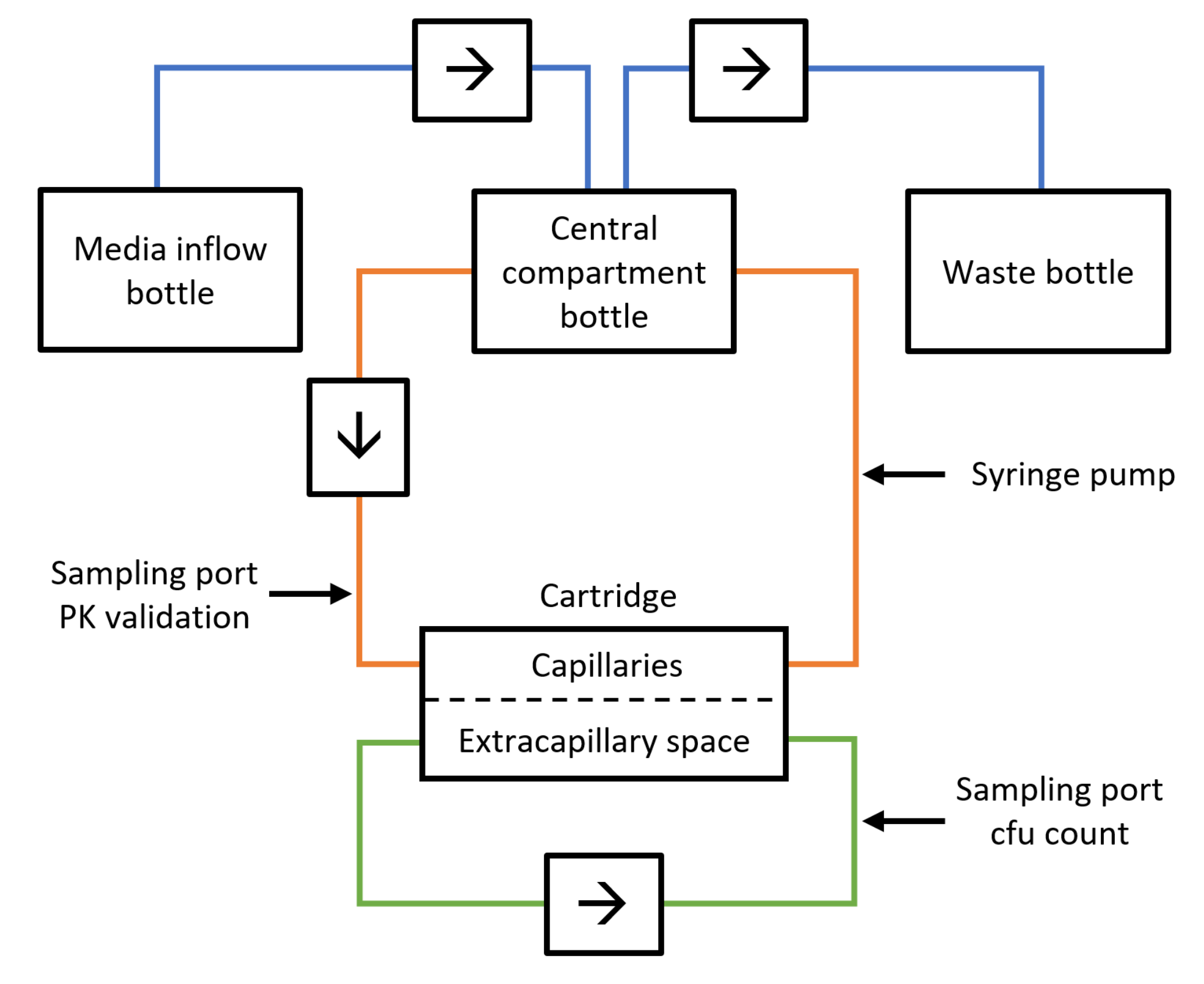
**

**Fig. S1.** Schematic overview of the hollow-fibre infection model used in this study. Boxed arrows indicate peristaltic pump placement and pump direction. Blue lines represent the tubing through which fresh media is continuously supplied to the central compartment and its contents are pumped out. Orange lines represent the tubing circuit connecting the central compartment and the cartridge. The cartridge fibres are semi-permeable allowing equilibration of nutrients and drug concentrations between the capillaries and the extracapillary space (i.e., the bacterial compartment). The contents of the extracapillary space are circulated in the opposite direction as the content of the central compartment, indicated with green lines. The locations of syringe sampling ports for PK validation and quantification of cfu as well as the syringe pump for drug infusion are also indicated.


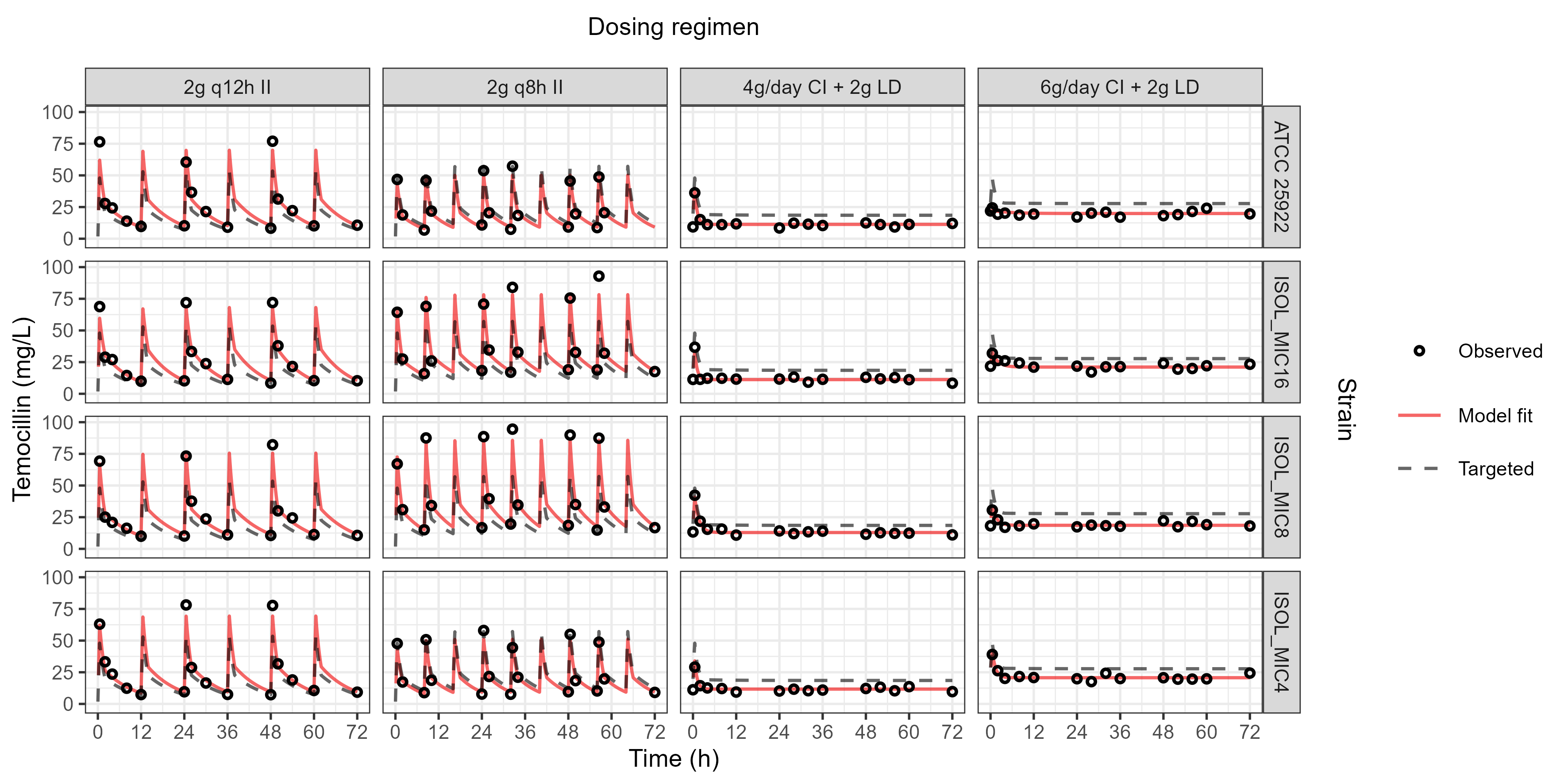


**Fig. S2.** Targeted and measured temocillin concentrations in the hollow-fibre infection model experiments, as well as the fit of the pharmacokinetic models describing the measured concentrations in each experiment. II: intermittent infusion; CI: continuous infusion; LD: loading dose.


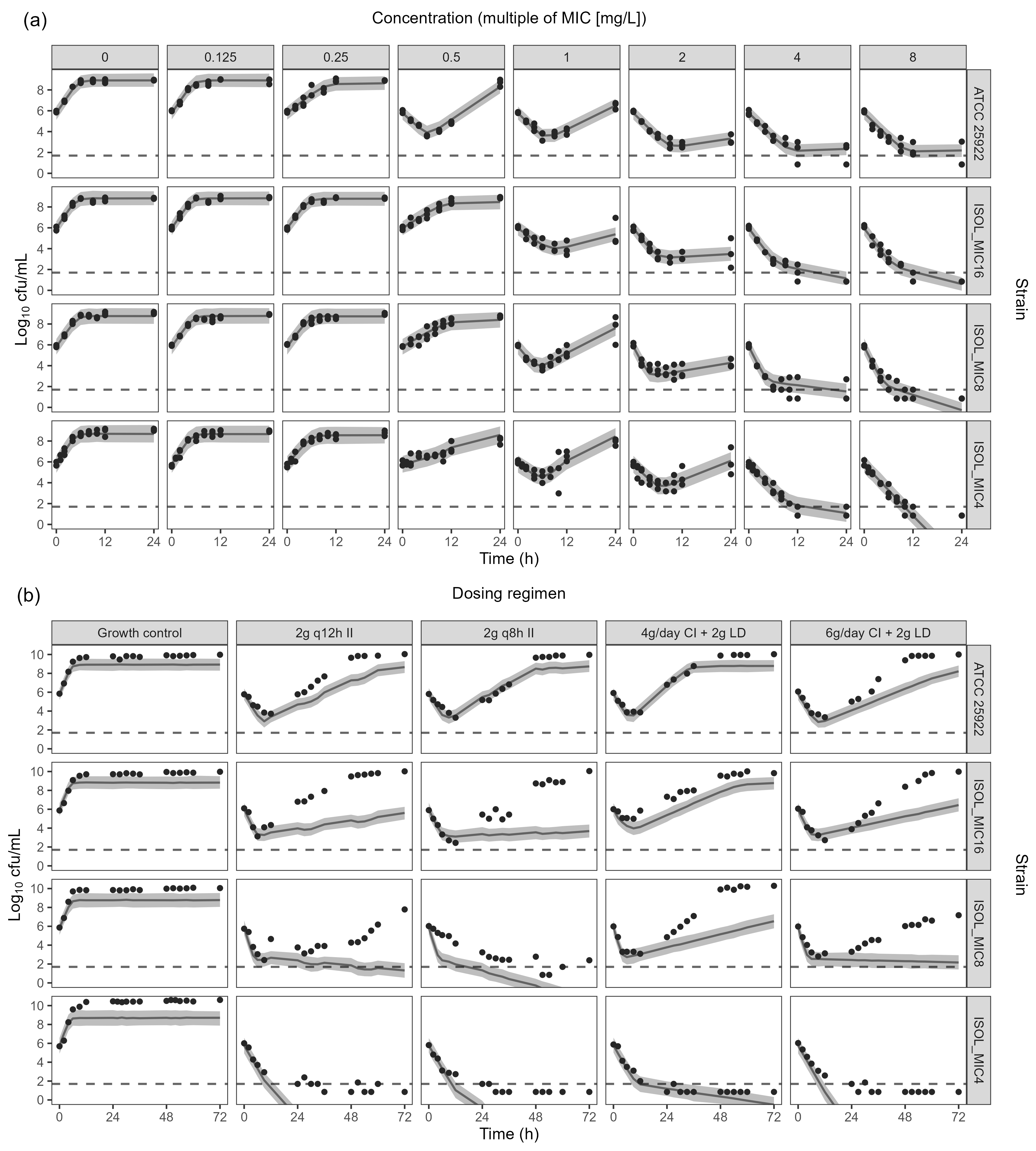


**Fig. S3.** Visual predictive check showing the fit of the pharmacokinetic-pharmacodynamic model developed using only static time-kill data to the observations in the static time-kill (a) and hollow-fibre infection model (b) experiments. Symbols represent observations for the total bacterial population. Solid lines represent the median values of model simulations and the shaded areas the 95% prediction intervals. The dashed line indicates the limit of detection (LOD; 50 cfu/mL). Observations below the LOD are plotted at log_10_(LOD)/2. II: intermittent infusion; CI: continuous infusion; LD: loading dose.


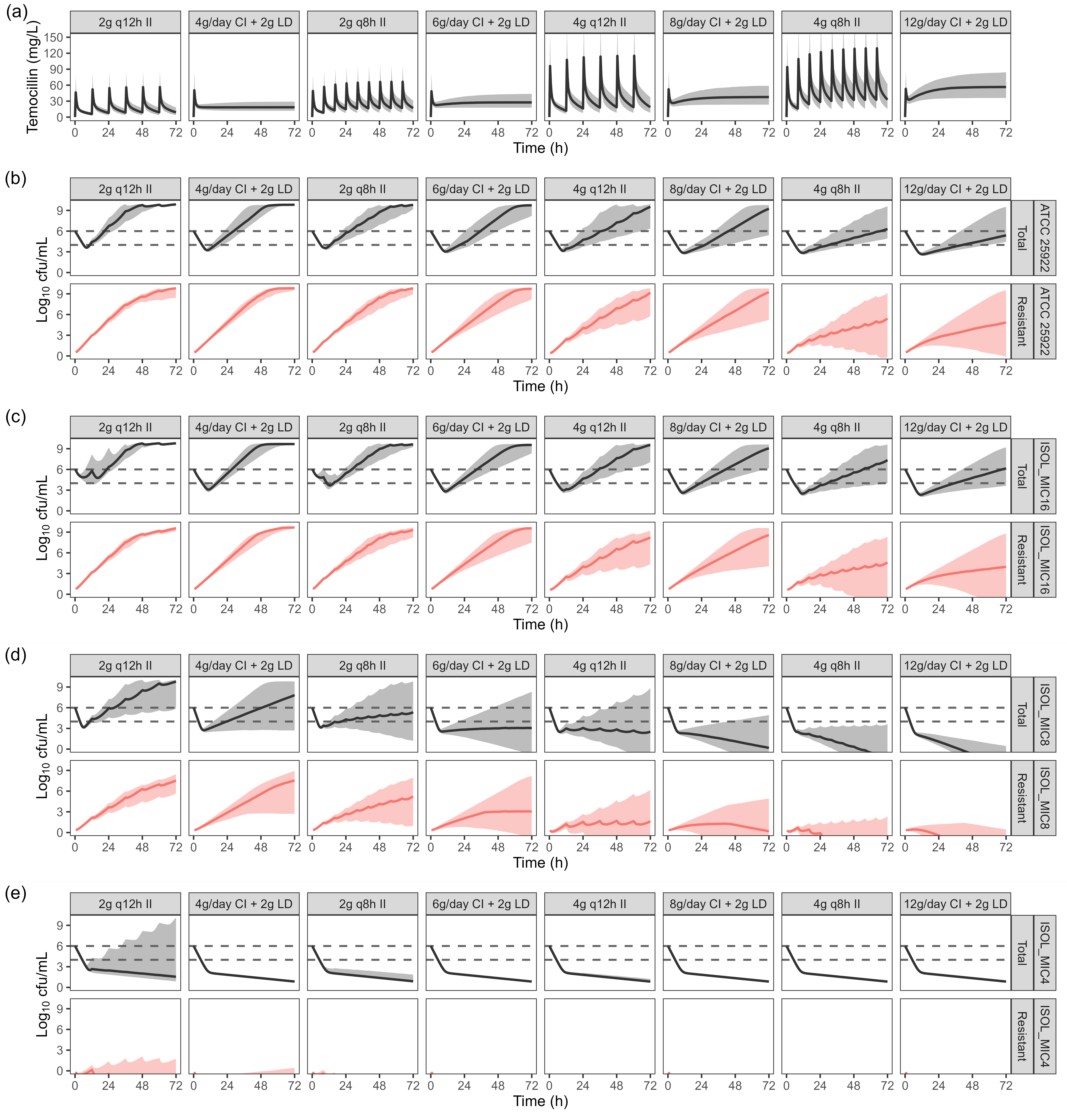
 **Fig. S4.** Predicted time-courses of unbound temocillin concentrations (a) and bacterial counts (b-e) of the total populations (black) and the resistant subpopulations growing on agar containing 32 mg/L temocillin (red) for the four different strains. The dosing regimens simulated in the four leftmost panels are currently in clinical use, while the four rightmost panels represent alternative regimens. The fraction unbound was set to 0.41. The solid lines represent the median values of model simulations (n=1,000). The shaded areas cover the 10^th^ and 90^th^ percentiles. The horizontal dashed lines correspond to the initial size of the total bacterial population (10^6^ cfu/mL) and a 2-log_10_ reduction in bacterial count. II: intermittent infusion; CI: continuous infusion; LD: loading dose.





**Fig. S5.** Predicted cfu/mL of the total (top panels in each plot a-h) and the resistant population (bottom panels) at 24 h (plots on the left) and 72 h (plots on the right) following different dosing regimens. Simulations were performed with the mean fraction unbound (fu) of 0.41 (results also shown in the main text), as well as the mean fu +/- one standard deviation (i.e. 0.16) reported by Laterre et al.^1^ The horizontal dashed lines correspond to the initial size of the total bacterial population (10^6^ cfu/mL) and a 2-log_10_ reduction. II: intermittent infusion; CI: continuous infusion; LD: loading dose.

**Validation of a HPLC-MS/MS assay for the determination of temocillin concentrations in CAMHB**

A previously described HPLC-MS/MS method to quantify total and unbound temocillin concentrations in serum^2^ was adapted and partially validated for determination of total temocillin concentrations in CAMHB in line with FDA guidelines.^3^ These FDA guidelines were also followed to evaluate trueness, precision, accuracy, limit of quantification (LOQ) and limit of detection (LOD), extraction efficiency and matrix effect (post-extraction addition technique). The calibration experimental design was 10 × 3 × 3 (calibrators of ten concentration levels, each replicated three times over three days). A 4 × 3 × 3 experimental design was used for the quality control (QC) samples. Data from calibrators were used to build the calibration curves (peak area ratios of temocillin/ticarcillin (internal standard, IS) versus concentration), and linear regression was used to predict the QC concentrations. Temocillin stock solutions were prepared in Ultrapure water (10 mg/mL) and diluted with CAMHB to prepare 10 calibrators (CS) (range = 0.99 - 478.19 mg/L). QC samples (0.99, 4.762, 24.937, and 196.078 mg/L) were prepared from an independent stock solution. Then, 200 μL of CS or QC was treated with 600 μL methanol after addition of 30 μL IS (1 mg/mL). After vortexing for 5 seconds, samples were centrifuged at 11,000 g, and 10 μL supernatant was injected into the HPLC/MS-MS. Statistics were performed using JMP software (SAS Institute, USA).

The correlation coefficient for each calibration curve was >0.9981 (Fig. S5). Relative bias was ≤-4.71%. The maximum relative standard deviation (RSD%) values for repeatability and highest intermediate precision were 4.13% and 4.52%, respectively. The method was considered accurate in the 0.99–478.19 mg/L range (Fig. S6). Extraction recovery ranged from 95.28% to 101.58%. The matrix effect was evaluated by comparing the slope and the intercept of the linear regression obtained with and without matrix. No significant difference was observed for the intercept (p=0.72) nor for the slopes (p=0.63) (Wilcoxon test, p > 0.05), indicating limited impact of matrix effect on the measured temocillin concentrations.


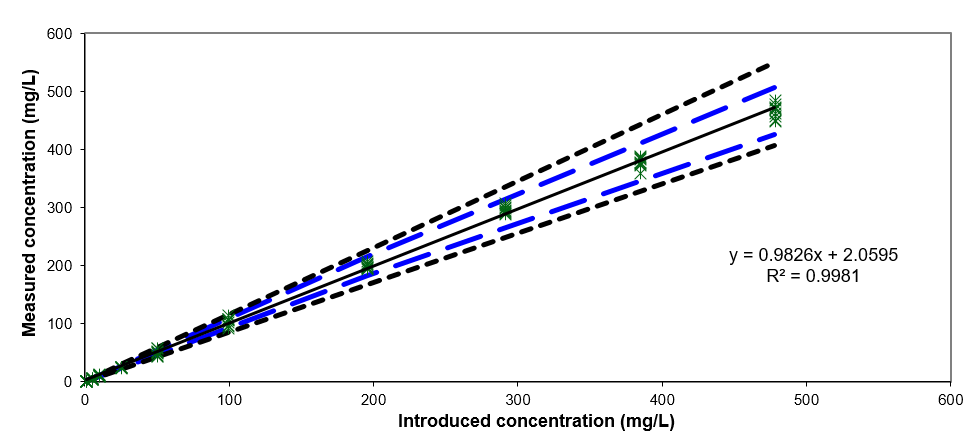


**Fig. S6.** Linear profile of temocillin in CAMHB. The solid line is the line of identity, the dashed blue lines represent the β-expectation tolerance limits (β=80%), and the dotted black lines represent the acceptance limits (±15% and ±20% for the LOQ). Symbols represent the relative back-calculated concentrations of the validation standards and are plotted according to their target concentration.


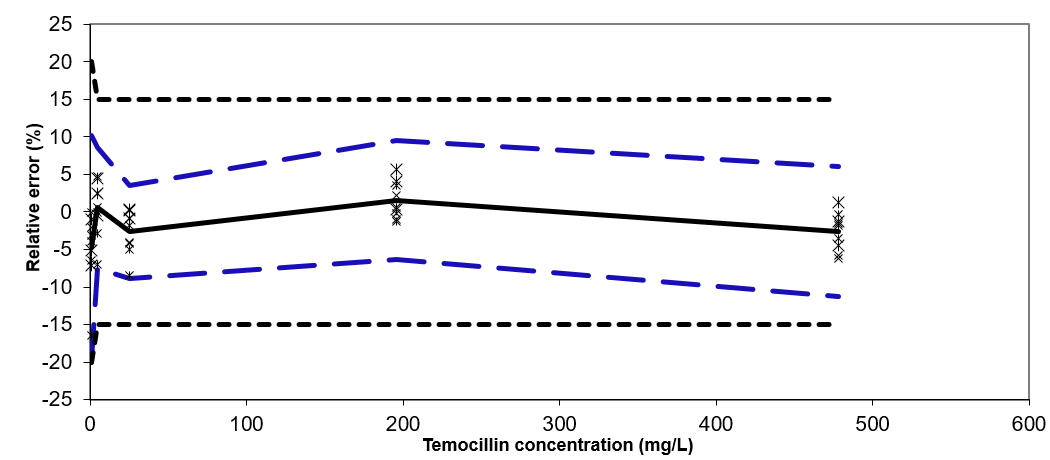


**Fig. S7.** Accuracy profile. The solid line is the relative bias, blue dashed lines represent the β-expectation tolerance limits (β=80%), and the dotted black lines represent the acceptance limits (±15% and ±20% for the LOQ). Symbols represent the relative back-calculated concentrations of QC samples.

**References**

1. Laterre PF, Wittebole X, Van de Velde S *et al.* Temocillin (6 g daily) in critically ill patients: continuous infusion versus three times daily administration. *J Antimicrob Chemother* 2015; **70**: 891-8.

2. Ngougni Pokem P, Miranda Bastos AC, Tulkens PM *et al.* Validation of a HPLC-MS/MS assay for the determination of total and unbound concentration of temocillin in human serum. *Clin Biochem* 2015; **48**: 542-5.

3. Food and Drug Administration. Bioanalytical Method Validation: Guidance for Industry. <https://www.fda.gov/files/drugs/published/Bioanalytical-Method-Validation-Guidance-for-Industry.pdf>.
